# Dusty: an assistive mobile manipulator that retrieves dropped objects for people with motor impairments

**DOI:** 10.3109/17483107.2011.615374

**Published:** 2011-10-21

**Authors:** Chih-Hung King, Tiffany L Chen, Zhengqin Fan, Jonathan D Glass, Charles C Kemp

**Affiliations:** 1 Healthcare Robotics Laboratory, Georgia Institute of Technology, Atlanta, GA, USA; 2 Emory ALS Center, Emory University, Atlanta, GA, USA

**Keywords:** Assistive robotics, manipulation aids, mobile manipulator, teleoperation, usability study

## Abstract

People with physical disabilities have ranked object retrieval as a high priority task for assistive robots. We have developed Dusty, a teleoperated mobile manipulator that fetches objects from the floor and delivers them to users at a comfortable height. In this paper, we first demonstrate the robot's high success rate (98.4%) when autonomously grasping 25 objects considered important by people with amyotrophic lateral sclerosis (ALS). We tested the robot with each object in five different configurations on five types of flooring. We then present the results of an experiment in which 20 people with ALS operated Dusty. Participants teleoperated Dusty to move around an obstacle, pick up an object, and deliver the object to themselves. They successfully completed this task in 59 out of 60 trials (3 trials each) with a mean completion time of 61.4 seconds (SD=20.5 seconds), and reported high overall satisfaction using Dusty (7-point Likert scale; 6.8 SD=0.6). Participants rated Dusty to be significantly easier to use than their own hands, asking family members, and using mechanical reachers (p < 0.03, paired t-tests). 14 of the 20 participants reported that they would prefer using Dusty over their current methods.

## Introduction

In 2005, the U.S. Census Bureau estimated that more than 3.3 million Americans have motor impairments [1]. People with motor impairments have consistently placed a high priority on the ability to retrieve out-of-reach objects, including objects on the floor [2]. Motor impairments can both increase the chances that an individual will drop an object, and make unassisted recovery of an object difficult or impossible. In a survey we conducted previously, 8 people with amyotrophic lateral sclerosis (ALS) reported dropping objects an average of 5.5 times a day with a self-reported mean object retrieval time of 9.4 SD 25.4 minutes [3]. The absence of a caregiver can lead to especially long recovery times, including one report of a 2-hour wait in our small week-long study [3].

**Implication for Assistive Technologies**

- A simple robot hand can effectively pick up a wide variety of objects relevant to people with motor impairments from common flooring.
- A relatively low cost, compact mobile robot can use a simple hand and a vertical lift to pick up and deliver dropped objects to people with motor impairments.
- In a laboratory setting, people with motor impairments were able to use a semi-autonomous mobile robot named Dusty to successfully pick up an object from the floor and deliver it.
- Participants reported high satisfaction with the robot Dusty, and on average they reported that it was easier to use than their current methods.

Robots could potentially help people with motor impairments retrieve dropped objects, and thereby gain greater independence. In this paper, we present a small-scale teleoperated mobile robot named Dusty, which is capable of reliably picking up objects from the floor and delivering them to motor-impaired users. Our results suggest that assistive mobile manipulators like Dusty could benefit users, and may be a practical assistive technology in the near term.

Within this paper we describe the design of the robotic system, the results of testing Dusty's grasping performance on high-priority objects, and the results of a user study with ALS patients from the Emory ALS Center. The results of the grasping experiments demonstrate Dusty's ability to grasp objects. Specifically, we tested Dusty's capabilities with the top 25 object categories ranked by people with ALS [3] under various configurations and on various types of flooring. The results of our user study show that participants can operate the robot to fetch and deliver an object robustly, safely and effectively. Participants also reported high satisfaction with Dusty and ranked the robot as significantly easier to use than their current methods of retrieving dropped objects.

## Related work

Currently, people with motor impairments retrieve dropped objects through a variety of means, including caregiver assistance, mechanical reachers, service animals and wheelchair-mounted robot arms (WMRAs). When people are unable to recover a dropped object on their own, enlisting the assistance of a caregiver is a common method for recovering the object. However, this requires that a caregiver be nearby and available, and it can diminish an individuals sense of independence.

A mechanical reacher is a common assistive technology that is comprised of a gripper, an adhesive pad, magnets or other mechanisms attached to the end of a long rod [4]. A user can manually manipulate the mechanical reacher to retrieve an object from the floor. Although using a mechanical reacher is a cost-effective solution, it requires the user to have significant dexterity and strength in his arms, hands and torso. In addition, the operating range is limited by the reaching distance of the device. Service animals, such as helper monkeys and service dogs, are trained to perform assistive tasks such as retrieving objects from the floor. However, service animals are expensive ($17,000-$35,000) [5], have long waiting lists, and require care ^[6]^ In addition, service animals may not be suitable for some users due to other conditions such as allergies [7].

WMRAs have been developed to assist wheelchair users in performing various tasks including object fetching and object delivery [8–12]. Researchers have also focused on improving the control interface for WMRAs [13–17]. There are commercially available WMRAs [18–20], and several studies have evaluated their performance with motor-impaired people [21–26].

However, commercially available WMRAs are expensive ($12,500–$50,000), and require the user to drive the wheelchair to the desired object in order for the arm to perform tasks. Users have reported that the size of WMRAs may hinder their ability to reach a table or maneuver the wheelchair through narrow passages [27]. Mobile manipulators have the potential to overcome these limitations since they are decoupled from a user's wheelchair and can move independently through an environment. This can allow users to continue using their existing wheelchairs. It also could enable the robot to assist those who do not require the use of a wheelchair, and may let users retrieve objects from places other than their current location. In addition, WMRAs may have difficulty grasping common objects from the floor, such as credit cards and small pills.

Several autonomous mobile robots have been developed to fetch and deliver objects to people [28–32]. However, very few of these robots have been evaluated directly with their target users [33–34]. CERO is a mobile robot with a flat top upon which objects could be placed for transport. It was evaluated in an office environment over a period of 3 months [35]. However, CERO does not have an arm or an end effector with which to grasp objects, and instead relies on people to place objects on it for delivery. In our prior research at the Healthcare Robotics Lab (HRL) at Georgia Tech, we developed the robot EL-E which could fetch and deliver an object that had been briefly illuminated with a laser pointer. We conducted user studies with people who have ALS in order to evaluate EL-E's performance in object fetching [36] and in object delivery [37] in two separate studies. However, we did not test EL-E's performance for object acquisition and delivery together. In our study, we found that delivering objects to a surface was more reliable compared with delivering an object directly to a user's hand [37]. Based on these findings, we designed Dusty so that it delivers objects to users on an elevated tray, which is an integral part of Dusty's end effector.

To date there has been little effort to develop compact and economical mobile manipulators to fetch and deliver objects. We have designed Dusty to be small in size, which makes it more portable and lighter weight than most human-scale mobile manipulators. This can be advantageous for using Dusty at multiple locations, shipping it to customers, safely interacting with it, and such. In addition, Dusty is less expensive to build than the current human-scale mobile manipulators. For example, in early 2011, the commercially available PR2 from Willow Garage was listed for $400,000 USD. In contrast, it costs less than $3000 USD of research-grade materials to fabricate Dusty, and the assembly process is straightforward. In the long run, the price of human-scale mobile manipulators may drop dramatically due to economies of scale and technological improvements. Human-scale mobile manipulators may also be able to perform a wider variety of assistive tasks by having a larger kinematic workspace, higher processing power and greater strength [32,37,38]. However, our results indicate that smaller, more specialized robots, like Dusty, may be able to provide affordable assistance in the near term, especially with tasks that involve small, lightweight objects.

We have previously published research related to Dusty at the IEEE International Conference on Robotics and Automation (ICRA) in 2009 [39], and the International Symposium on Quality of Life Technology (QoLT) in 2010 [40]. At ICRA, we presented the design and initial evaluation of the first version of Dusty's end effector. At QoLT we presented the current simplified design of Dusty, results from a pilot test of its grasping performance, and results from a pilot user study with able-bodied subjects. In this journal article, we present a new and more thorough evaluation of Dusty's grasping performance, and a new study involving 20 people with ALS representative of the intended end user population.

## System description

Dusty consists of a mobile robot with a joystick interface. A person uses the joystick to control the motion of the robot and pushes buttons to command the robot to grasp an object from the floor and lift the object up for delivery. The following sections describe the key features of the system.

### Robot dusty

**Figure 1:**
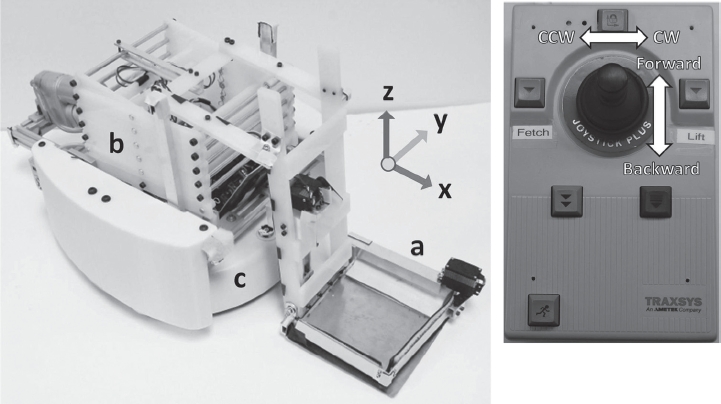
Mobile manipulator system. Left: The robot Dusty consists of (a) the end effector, (b) the lift, and (c) the mobile base; Right: the joystick interface consists of a joystick and two buttons labeled *Fetch* and *Lift*. The remaining buttons are non-functional.

The mobile robot comprises three main components: a mobile base, an end effector and a lift (Figure 1). The mobile base is an iRobot Create which receives information from the joystick interface through a Bluetooth-to-serial adapter (Ro-boDynamics RooTooth). The mobile base then relays control signals to an Arduino board which controls servo motors and a linear actuator which move the end effector and the lift, respectively. The following two subsections describe the end effector and the lift in more detail.

**Figure 2:**
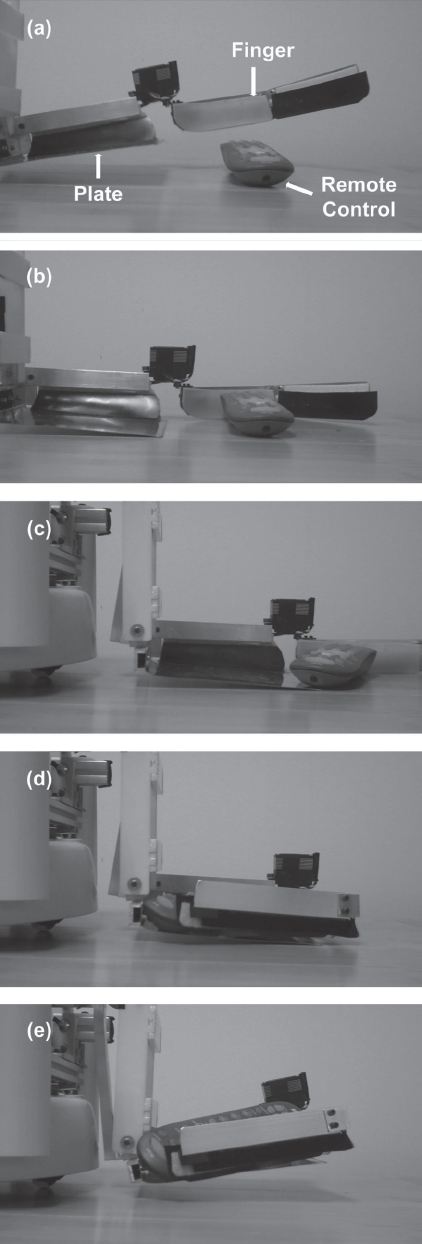
Sequence of the end effector grasping a remote control on a wooden floor: (a) the finger opens; (b) the plate tilts downward and the leading wedge touches the floor; (c) the robot moves forward and the plate slides underneath the object; (d) the finger closes, pushing the object into the end effector; (e) the plate tilts back to its original position.

The end effector consists of a square metal plate with a leading wedge that slides under an object and a finger that sweeps the object onto the plate. The plate is a 15 cm × 15 cm square steel sheet. We machined the front edge of the plate to be a wedge (i.e. the leading wedge). A servo motor (Hitec HS-7955TG, Hitech Inc., Poway, CA, USA) tilts the plate downward to a constant angle so that the wedge stops at the surface of the floor and presses down on it firmly. Because the thin plate is compliant and the wedge is a straight line segment, the wedge can make contact across its entire length with flat surfaces at a variety of heights. When the plate is at its resting angle (i.e. tilted slightly upward), it is held ∼1 cm above the floor (see Figure 2e). Another servo motor rotates the rigid, aluminum L-shaped finger. After this finger sweeps an object onto the plate, it is held closed in order to help secure the object. In this configuration, it covers the leading wedge, and serves as a fence along the front and right edges of the plate. As a result, the object is completely fenced in.

After the robot has used its end effector to pick up an object, the user can activate the robot's lift which raises the object to a predetermined height for the user to retrieve the object. The scissor lift is made of acrylic. Steel rods connect the corresponding joints on the two sides of the scissor lift. A linear actuator (ServoCity HDLS, Robotzone, LLC. Winfield, KS, USA) on the mobile base connects to the bottom of the scissor lift and moves one of the steel rods in order to extend the lift. When the lift is fully extended, the end effector is raised to a height of 74 cm above the floor, which is within the guidelines for tables and counters provided by the Americans with Disabilities Act [41].

### Joystick interface

The joystick interface uses a commercially available joystick (Traxsys Roller Plus Joystick, Traxsys Input Products, London, UK) that is designed to improve computer access for people with disabilities. The joystick connects to a computer that wirelessly communicates with the robot over Bluetooth. When the user moves the joystick to the left or right, the robot rotates counterclockwise or clockwise respectively (Figure 1). When the user moves the joystick forward or backward, the robot moves forward or backward, respectively. There are also two functional buttons on the joystick: the button labeled *Fetch* which activates the one-touch-and-grasp behavior (discussed in Section “One-touch-and-grasp behavior”), and the button labeled *Lift* which raises and lowers the end effector's tray. The latency between the joystick control and the robot's motion is around 230 ms. For this work, we affixed the joystick to an adjustable stand and placed it next to a seated participant. This implementation is representative of a wheelchair-mounted joystick.

### One-touch-and-grasp behavior

We have implemented an autonomous behavior that we call one-touch-and-grasp, which reduces the need for the user to control the robot in detail. The user first coarsely positions the robot in front of an object and then presses the *Fetch* button on the joystick. Dusty then performs the following autonomous behavior (see Figure 2): (1) the finger of the end effector opens; (2) the end effector tilts downward, so that its leading wedge touches the floor; (3) the robot moves forward toward the object for a duration of 2 seconds (at a speed of approximately 15 cm/s); (4) the finger closes, sweeping the object onto the end effector and securing it there; and (5) the end effector tilts back to its original angle. The user can press the *Fetch* button again in order to stop the behavior anytime after it has been initiated.

### Safety

Since we designed Dusty to operate in close proximity with people, we implemented several safety features. First, the robot has the following features to reduce contact forces: (1) the robot moves at a low velocity (<33 cm/s); (2) the robot (8.2 kg) and its end effector (1.1 kg) are relatively low mass compared with human-scale robots [27,31,36]; and (3) the fully extended lift and the link that connects the end effector to the lift have intrinsic compliance (104.2 N/m) in the robot's x-axis (refer to Figure 1). For example, when the extended end effector is displaced by 1.0 cm in the x direction, such as due to contact during delivery, it only applies about 1 N of force. Second, we covered the sharp edges of the robot's base with a smooth casing made out of ABS plastic, and covered sharp corners on the robot's frame with adhesive foam. We also covered one side of the finger with strips of rubber so that the leading wedge is covered with the rubber when the finger is closed. Third, we implemented an run stop feature that enables an experimenter to stop the robot's motion by pressing any key on the computer keyboard. During this study, an experimenter was always prepared to press a key if undesirable contact with the robot were observed. Fourth, when the end effector is raised, pressing the *Fetch* button will only open or close the finger on the end effector without moving the mobile base. This prevents the robot from moving forward unexpectedly when the lift is extended.

## Grasping performance of the end effector

**Figure 3:**
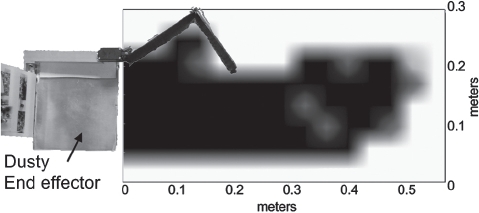
Results of grasping a small cylindrical object at points on a grid. Black represents a grasping success rate of 3/3, dark blue represents 2/3, light blue represents 1/3, and white represents 0/3.

In practice, the actual usefulness of Dusty would depend on its ability to easily grasp objects dropped by the user. We evaluated Dusty's grasping performance under variations of several important characteristics, including the type of object, the configuration of the object and the type of flooring upon which the object is resting. Preliminary testing showed that the end effector achieved a high success rate (>97%) grasping objects on a short-pile carpet, and could successfully grasp a small cylindrical object over a large rectangular area (50 cm × 12.86 cm, shown in Figure 3) [40].

**Figure 4:**
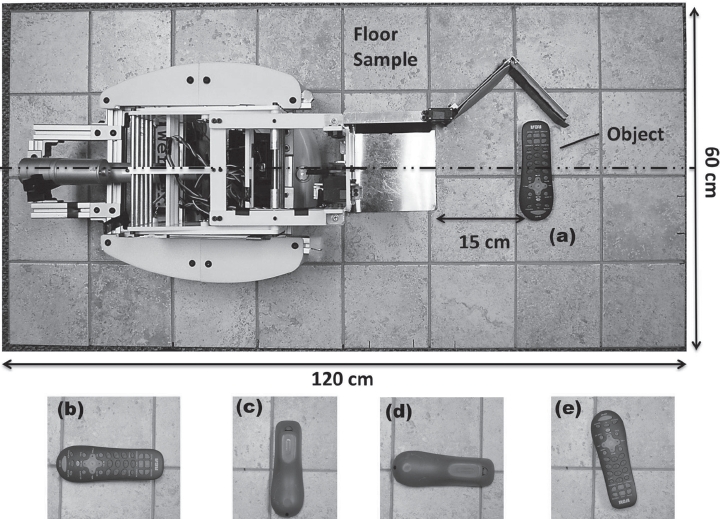
Experimental setup of the grasping tests: The object was placed 15 cm directly in front of the robot, and (a) to (e) show the 5 variations of the object's orientation.

In this experiment, we tested objects from 25 object categories ranked most important for robotic retrieval by people with ALS [3]. We have previously found that the more highly ranked objects tended to be smaller and lighter-weight, so we designed Dusty accordingly [3]. We selected five representative object configurations that included four variations in the objects orientation (Face/Parallel, Face/Perpendicular, Back/ Parallel and Back/Perpendicular; see Figure 4a-d). The fifth configuration could be any remaining degree of freedom (DoF) of the object. For example, we tested a pair of eyeglasses with the frame's legs folded for the first four configurations, and with the legs extended for the fifth configuration. When the object did not have additional DoF, we arbitrarily selected an orientation by dropping the object from a distance of∼10 cm above the floor surface (Figure 4e). Since homes have different types of flooring, and since we believe that the end effector's performance critically depends on the ability of the leading wedge to make contact with the floor, we tested the end effector on five different types of flooring. They include both hard (wood, granite, tile) and soft surfaces (short pile and medium pile). We expected that the end effector might perform better on soft surfaces, since the flat plate can compress the surface when sliding underneath the object.

For each grasp attempt, we placed each object 15 cm in front of the end effector (Figure 4), and then pressed the *Fetch* button to execute the one-touch-and-grasp behavior. We deemed a trial to be successful if the object was more than halfway on the plate after the finger closed and the robot stopped moving. Overall, we tested 25 objects in five configurations on five types of flooring, resulting in a total of 625 grasp attempts (25 × 5 × 5).

Our results show that over the 625 trials, the end effector successfully grasped objects 615 times, achieving an overall success rate of 98.4% (see Table I). The robot performed best on short-pile carpet (100% success), followed by medium pile carpet (99.2%), wood (98.4%), granite (97.6%) and ceramic tile (96.8%). The robot successfully grasped each object at least 22 out of 25 times (≥92%).

**Table I:** Results of object grasping tests on various flooring.

Rank^a^	Object class^a^	Granite	Wood	Ceramic	Short-pile carpet	Medium pile carpet	Success rate (%)
1	TV Remote	5/5	5/5	5/5	5/5	5/5	100
2	Medicine pill	5/5	5/5	5/5	5/5	5/5	100
3	Cordless phone	5/5	5/5	5/5	5/5	5/5	100
4	Prescription bottle	5/5	5/5	5/5	5/5	5/5	100
4	Fork	5/5	5/5	5/5	5/5	5/5	100
6	Glasses	5/5	5/5	5/5	5/5	5/5	100
7	Toothbrush	5/5	5/5	5/5	5/5	5/5	100
8	Spoon	5/5	5/5	5/5	5/5	5/5	100
9	Cell phone	5/5	5/5	5/5	5/5	5/5	100
10	Toothpaste	5/5	5/5	5/5	5/5	5/5	100
10	Book	5/5	5/5	4/5	5/5	5/5	96
10	Hand towel	5/5	5/5	5/5	5/5	5/5	100
13	Small envelope	5/5	5/5	4/5	5/5	5/5	96
14	Cup/Mug	5/5	5/5	5/5	5/5	5/5	100
15	Soap	5/5	5/5	5/5	5/5	5/5	100
16	Disposable bottle	4/5	5/5	5/5	5/5	5/5	96
17	Shoe	4/5	4/5	4/5	5/5	5/5	88
17	Dish bowl	5/5	5/5	5/5	5/5	5/5	100
19	Keys	5/5	5/5	5/5	5/5	4/5	96
20	Dish plate	5/5	5/5	5/5	5/5	5/5	100
21	Pen/Pencil	4/5	5/5	5/5	5/5	5/5	96
22	Table knife	5/5	4/5	4/5	5/5	5/5	92
22	Credit card	5/5	5/5	5/5	5/5	5/5	100
24	Medicine box	5/5	5/5	5/5	5/5	5/5	100
24	Bill	5/5	5/5	5/5	5/5	5/5	100
Success rate		97.6%	98.4%	96.8%	100%	99.2%	98.4

a Twenty-five object categories ranked most important for robotic retrieval by motor-impaired users from the Emory ALS Center in our previous study [3].

Seven failures occurred due to less than half the object being on the plate, and may relate to the finger moving the objects in the negative y direction (refer to Figure 1a). These seven failures were for a disposable bottle (1 attempt), shoe (3 attempts), table knife (2 attempts) and a small envelope (1 attempt). Another failed grasp occurred when the finger moved over a pen. Two other failures occurred when the plate inserted itself between the pages of a book and between keys on a key chain.

## Methodology of the user study

We conducted a user study with ALS patients at the Emory ALS Center to evaluate Dusty's performance in a representative task. In preparation for tests with people with motor impairments, we first conducted a pilot study with ten able-bodied lab members and used the results to refine the robot's design and the test protocols [40]. In this section, we describe the methodology of our user study beginning with participant recruitment. Next we describe the experimental design and setup. Lastly, we describe the procedure for each trial.

### Participants

We recruited 21 people with ALS for the user study, and excluded one of these participants because of an error in our experimental procedure. Table II shows the demographic information of the 20 participants. In total, eight participants reported that they had previously used or interacted with robots, five reported that they had played with robotic toys; two reported that they had interacted with the robot EL-E; and one reported that he had used a robot vacuum. ALS, also known as Lou Gehrig's disease, is a progressive neurodegen-erative disease that is characterized by the gradual degeneration of motor neurons [42]. As a result, an individual gradually loses his motor function. Amyotrophic Lateral Sclerosis Functional Rating Scale (ALSFRS) is an instrument for evaluating the functional status of patients with ALS [42]. Our participants' impairments were diverse, and we expect that our results would generalize to other populations with physical disabilities. We visited the Emory ALS Center a total of six times to perform experiments from April 16 to July 16, 2010 in conjunction with the Emory ALS Clinic. As the nurses at the ALS Center did their rounds, they asked patients whether they would be interested in participating in the study. The nurses limited participation to patients who had some functionality in at least one of their hands and one of their arms so that they would be able to operate the robot's joystick and reach for the object. If a patient agreed to participate, one research staff member from Emory and one experimenter from Georgia Tech obtained informed consent from the patient. Then, the experimenter from Georgia Tech asked the patient for demographic information and administered the pre-task survey. After completing the pre-task survey, the patient continued with his medical appointments at the ALS Center. After his appointments were completed, the experimenter led the patient to a room where the experiment was conducted.

**Table II:** Demographic information.

Gender	Male (15), Female (5)
Education past high school (year)	0–7 (2.1 SD = 2.1)
Ethnicity	White (17), African American (2), Hispanic (1)
Age (year)	38–77 (54.5, SD = 10.1)
Marital status	Single (3), Married (17)
ALSFRS score	23–40 (32.7 SD = 5.2)
Used a robot before	8/20 (40%)
Own a powered wheelchair	10/20 (50%)

### Object retrieval task

We asked participants to control Dusty and perform an object retrieval task. We designed the retrieval task to simulate a situation in which a motor-impaired person has dropped an object from a seated position and wishes to retrieve it. The person would then use the robot to retrieve the object by first driving the robot from its current location in the room to the object while avoiding obstacles, such as furniture. Then the user would command the robot to pick up the object and deliver the object.

**Figure 5:**
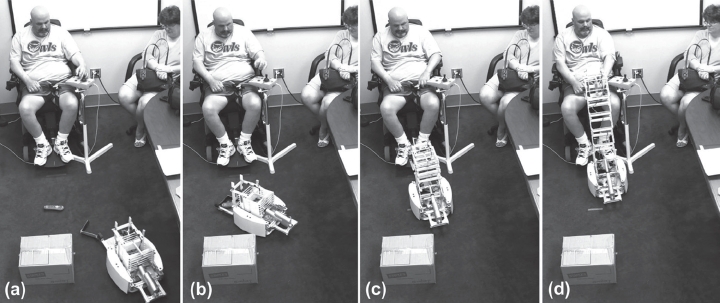
Object retrieval task: This figure illustrates how a participant with ALS used Dusty to retrieve a remote control from the floor while seated in a PWC: (a) navigate around the obstacle; (b) press the *Fetch* button which commands the robot to pick up the object; (c) navigate close to himself and press the *Lift* button; and (d) grab the object at a comfortable height (IRB approval and user permission obtained).

For example, the participant in Figure 5 used the joystick to drive the robot toward him and navigate the robot around an obstacle. He then navigated the robot toward an object placed in front of his chair, and pressed the *Fetch* button on the joystick to command the robot to autonomously grasp the object. He pressed the *Lift* button and then drove the robot closer to him, so that he could reach the object. In some trials, people first drove the robot closer and then pressed the *Lift* button.

### Experimental setup

**Figure 6:**
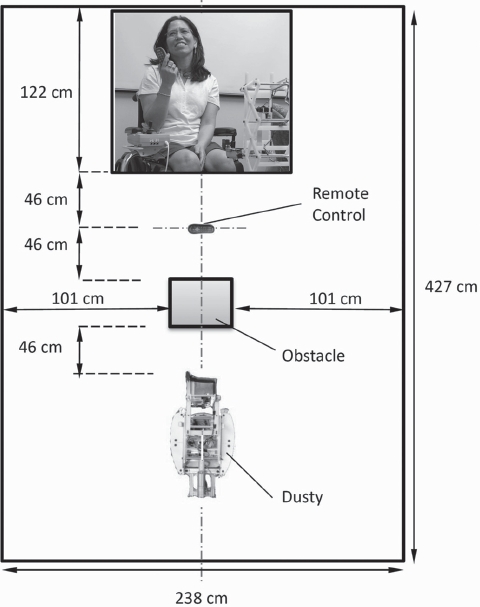
Experimental setup of the user study: Dusty is in front of the participant, and an obstacle is placed between the participant and the robot (IRB approval and user permission obtained).

We conducted the study in a room at the Emory ALS Center with short-pile carpet in a space measuring 2.4 m × 4.3 m (Figure 6). Each participant was seated for the entire experiment, which took around 35 minutes to complete. When a participant brought his own wheelchair, he sat in it for the study. When a participant came without a wheelchair (11 out of 20), we provided a desk chair for him to use. We positioned the participant's chair so that his back was facing a wall and his feet (or the wheelchair footrests) were at a distance of 1.2 m away from the wall (see Figure 6). We also consistently positioned the remote control, the obstacle and the robot as shown in Figure 6. We adjusted the position and height of the joystick stand to ensure that each participant was comfortable with the placement of the joystick interface. Then, we explained how to use the joystick and buttons to control Dusty and gave each participant 3 minutes to become familiar with using the joystick interface and performing the object retrieval task. As part of the training, we taught the participant how to orient the end effector with respect to the object before pressing the *Fetch* button.

Each participant performed the object retrieval task (Figure 5) 3 times. We considered a trial successful if the participant was able to grasp the object from the end effector and place the object in his lap. For all trials, we used a TV remote control as the target object for retrieval because it ranked as the number one object category in our prioritized object list [3]. We placed the remote control 45 cm in front of the participant because we anticipated that dropped objects would fall close to the user (Figure 6). We used a cardboard box (36 cm × 28 cm × 22 cm) as the obstacle placed between the participant and the robot, and placed it 90 cm from the participant and 46 cm from the robot.

We recorded the following objective data during each trial: time to complete the task, number of times that Dusty collided with the obstacle, number of times that Dusty failed to grasp the object, number of times that the object dropped from the end effector during delivery, number of times that Dusty made contact with the wheelchair or chair, and the number of times that Dusty made contact with the participant. We defined a grasping attempt as successful if the object was on the plate of the end effector after the robot completed the one-touch-and-grasp action.

### Surveys

We administered a demographic survey, a pre-task survey and a post-task survey. In the pre-task survey, we asked participants to estimate the number of times per day that they drop objects. We asked participants to select the methods they currently use to retrieve dropped objects from a list. The list contained the following methods: “Ask family members,” “Ask nurses/caretakers,” “Use my hands,” “Use mechanical reachers (grippers, sticky rods),” “Service animals,” “Assistive robots/WMRAs,” and “Others (Specify).” In this paper, we refer to these as *asking family members, asking caregivers, using their hands, using reachers, service animals, robots* and *others*, respectively. We also asked participants to score the ease of use for each of the methods they reported using.

We conducted a post-task survey for each participant upon completion of the object retrieval tests. We asked participants whether they would prefer using Dusty over the methods they reported using in the pre-task survey. We also asked participants whether they would use Dusty in various rooms typically found in a home including the bedroom, living room/family room/den, kitchen, bathroom, dining room, home office/reading room/play room, porch/balcony and other. We also administered a questionnaire asking participants about their perceptions of Dusty's performance and various design attributes. Questions 1 through 14 are measured on a 7-point Likert scale, where 1 = “Strongly disagree,” 4 = “Neutral,” and 7 = “Strongly agree.” The last two questions are binary.

1. I am satisfied with the time it took to complete the task using the robot.
2. I could effectively use the robot to retrieve the object from the floor.
3. It was easy to navigate the robot around obstacles.
4. I could effectively control the robot to grab the object from the floor.
5. I could easily retrieve the object from the robot's hand.
6. Any delay between the joystick control and robot's move ment did not hinder my ability to perform the task.
7. It was fun to control the robot.
8. I was worried that I might break the robot.
9. I was worried about my safety.
10. I am satisfied with the speed that the robot was moving.
11. I am satisfied with the usability of the interface.
12. I am satisfied with how the robot looks.
13. The robot made it easy to retrieve the object from the floor.
14. Overall, I was satisfied using the robot.
15. Are you satisfied with the height of the lift?
16. Are you satisfied with the size of the robot?

## Results

Table III shows the results of the object retrieval trials. Participants successfully used Dusty to retrieve the object 59 out of 60 times, achieving an overall success rate of 98%. All participants were able to complete the task in at least two out of the three trials. The overall mean completion time for the successful trials was around 1 minute (61.4 SD = 20.5 seconds). Although the mean completion time decreases from trial 1 to trial 3, the decrease was not significantly different across all three trials (p >0.2, paired t-tests). We also did not find a significant correlation between the completion time and the ALSFRS scores (R^2^ = −0.20). There was no example of the object dropping from the end effector during the test.

**Table III:** Results of the object retrieval test.

Completed trials		59/60 (98%)
	Trial 1	65.4 SD = 21.8
	
	Trial 2	60.4 SD = 20.6
	
	Trial 3	58.2 SD = 19.4
	
Mean task completion time (sec)	Overall	61.4 SD = 20.5

Contact with obstacle		23^a^
Failed grasping attempt		13^a^
Dropped object		0
Contact with participant		2
Contact with wheelchair		3

a Nine attempts occurred during the failed trial.

During the one failure case, the participant navigated the robot close to the object but did not orient the end effector towards the object. Consequently, the robot moved forward and failed to grasp the object after the participant pressed the *Fetch* button. The participant then navigated the robot backward, causing the robot to hit the obstacle. The participant continued to operate the robot in this pattern (misorienting the end effector, failing to grasp the object, moving the robot and hitting the obstacle) 9 consecutive times. After more than 3 minutes (199.4 seconds) into the experiment, the participant decided to abort the trial. However, the participant successfully completed the first trial in 66.9 seconds and the third trial in 71.6 seconds.

The robot made contact with two participants (Table III). In one case, the participant navigated the robot so that the end effector made contact with his shoe. In the second case, while the scissor lift was fully extended upward, the participant navigated Dusty so that the end effector made contact with his thigh. Neither of the participants reported injury or discomfort when the robot made physical contact. In general, peripheral sensory neuropathy is not considered to be a feature of ALS [43]. In addition, there were three cases where Dusty made contact with the footrests of participants' wheelchairs. Since all of these contact occurrences were brief and happened while the robot was moving at a slow velocity, the experimenter did not press the emergency stop key on the computer and did not terminate the trial.

**Figure 8:**
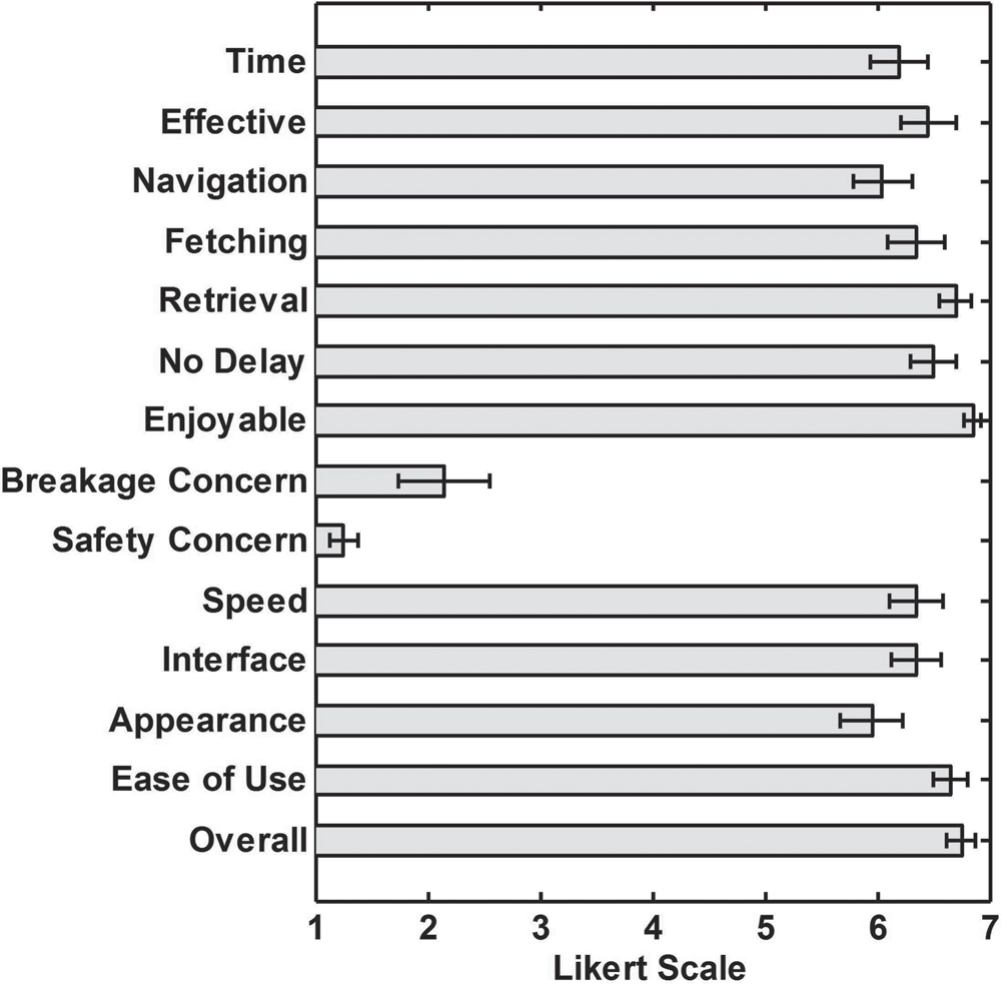
Participants' subjective responses about Dusty's features and performance: Questions 1–15 in Section “Surveys” 5.4. (7-point Likert scale; 1= Strongly disagree; 7 = Strongly agree). Error bars show the standard error of the mean.

Participants reported high satisfaction with Dusty's performance and features. They reported Likert scale responses on average greater than a score of 6 = “agree” for questions 1–7, and questions 10–14 (refer to Section “Surveys” and Figure 8). Participants also reported little concern about their safety (1.3 SD = 0.5) or breaking the robot (2.2 SD = 1.8). 19 participants (95%) were satisfied with the height of the lift, and one participant wanted the lift to be 0.75″ to 4″ higher, possibly due to him being seated higher with his powered wheelchair. 18 participants (90%) reported to be satisfied with the size of the robot, and 2 participants wanted Dusty to be 20–30% smaller. Overall, participants reported very high satisfaction with Dusty (6.8 SD = 0.6; range: 6–7).

Table IV shows the results of pre-task and post-task surveys for the methods the participants currently use to retrieve dropped objects. 17 participants reported *using their hands* to retrieve dropped objects, followed by *asking family members* (12 participants), *using reachers* (three participants) and *asking caregivers* (one participant). One participant reported using a homemade rod with a hook and a rod with a magnet. In the analysis we categorized these two methods as *using reachers* which in turn has a total of five responses. No participant reported using *robots* or *service animals*. We performed statistical comparisons among the object retrieval methods that had more than two responses. As a result, we compared the participants' responses for *using their hands, asking family members* and *using reachers*. The percentage of time they reported *using their hands* was significantly higher than the percentage of time they reported *asking family members* (p <0.01, paired t-tests). There was no significant difference for the reported retrieval time among the 3 methods. Participants reported that Dusty is significantly easier to use compared with the 3 alternate methods (Figure 9, p < 0.03, paired t-tests).

**Figure 7:**
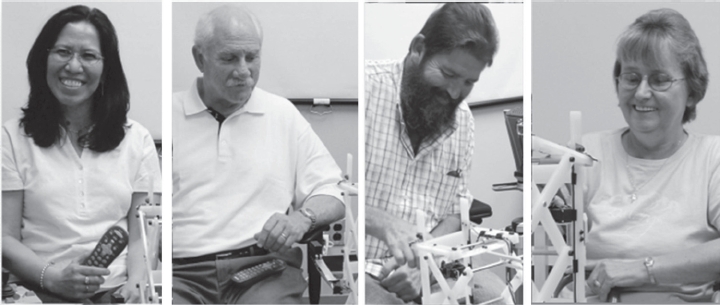
Facial expressions of subjects after successfully retrieving the object: Qualitatively, we observed positive expressions from the majority of participants upon retrieving the object (IRB approval and user permission obtained).

**Figure 9:**
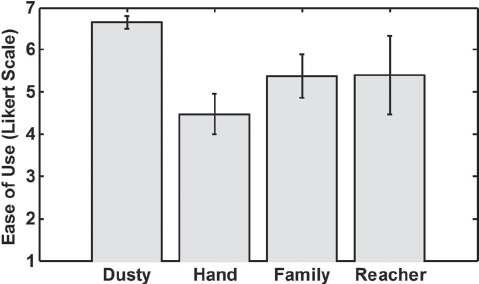
Reported ease of use of Dusty compared with participants' responses about their current retrieval methods: (7-point Likert scale; 1 = Strongly disagree; 7 = Strongly agree). Error bars show the standard error of the mean. The bar representing Dusty is significantly higher than the rest of the bars (p < 0.03, paired t-tests).

**Table IV:** Characteristics of dropped object retrieval methods reported by participants.

	Pre-task survey		Post-task survey
		
Method	Number of responses	Mean ease of use (7-point Likert)	Prefer using Dusty
Hand	17	4.5 SD = 2.0	12/17 (71%)
Family member	12	5.4 SD = 1.8	11/12 (92%)
Readier	5	5.4 SD = 2.1	5/5 (100%)
Caregiver	1	7	1/1

In the post-task survey, we asked participants to compare using Dusty with what they reported to be their current methods of retrieval. Fourteen participants (70%) responded that they would prefer using Dusty over all of their current methods. Twelve out of the 17 participants (71%) who reported *using their hands* responded that they would prefer using Dusty (Table IV). Five participants responded that they wanted to keep *using their hands* while they still had the ability to do so, but would prefer using Dusty if their disease progression compromised this ability. Eleven out of the 12 participants (92%) who reported *asking family members* responded that they would prefer using Dusty, and one responded that she would prefer *asking family members* because of easy access. All five participants who reported *using reachers* responded that they would prefer using Dusty. The participant who reported *asking caregivers* also responded that he would prefer using Dusty.

Figure 9 shows the percentage of participants who reported that they would use Dusty in each type of room. Over 85% of participants expressed that they would be willing to use Dusty in all the rooms of their house except for the bathroom.

Participants of 60% reported that they would be willing to use Dusty in the bathroom. The primary reason participants stated for not wishing to use Dusty in the bathroom was limited space, which accounted for five of the eight responses. In addition, limited space was the most common reason given for not using Dusty in any type of room (6 responses), followed by participants not spending time in the room (4 responses).

Table V compares responses from the 10 participants who owned powered wheelchairs (PWC) and the ten who did not. Our results show that the PWC owners had significantly lower ALSFRS scores, reported significantly higher rates of dropping objects, and reported using significantly more methods to retrieve objects from the floor. All PWC owners reported *asking family members* to retrieve dropped objects, whereas all of those who did not own a PWC reported *using their hands* to retrieve objects. Interestingly, we found no significant difference between the mean task completion time for participants who owned a PWC and for those who did not (p = 0.15, paired t-tests).

**Table V:** Comparison between participants who own and do not own a powered wheelchair (PWC).

Measurement	PWC(*n* = 10)	no PWC (*n* = 10)	p-value
ALSFRS Score	29.9 SD = 5.4	35.4 SD = 3.2	p = 0.01
Reported no. of dropped objects per day	4.9 SD = 3.9	1.6 SD = 1.4	p = 0.02
Reported no. of retrieval metohds	2.3 SD = 0.8 methods	1.2 SD = 0.4	p < 0.01

Unexpectedly, there were two participants in this study who had also participated in a previous object fetching study with the robot EL-E [37]. During the debriefing, both participants indicated that they would prefer using Dusty over EL-E around the house. After the experiment was over, we called these two participants and asked them to elaborate on their experiences with both Dusty and EL-E. We gained permission from one participant to record the phone call, and these are some of the transcribed quotes: “EL-E is tall and cumbersome to have around the house”; “Dusty is less bulky and I can bring it everywhere I go”; “Dusty is more practical”; “Dusty can go under the table”; and “Dusty is much faster to use to pick up objects”.

## Discussion

Dusty's high success rate in the grasping experiments (98.4%) demonstrates the robustness and effectiveness of our end effector design. The end effector is effective in grasping high-priority objects which vary in size, shape and weight, and is effective on hard surfaces (granite, ceramic and tile, >96%) and on soft surfaces (short-pile and medium-pile carpets, >99%). The results suggest that the end effector is a viable solution for fetching dropped objects in real environments. We found that in addition to the leading wedge making good contact with the floor, having good contact between the finger and the floor was important. In the trials when the object was on a hard surface, closing the finger typically moved the object onto the plate, even though the plate could not slide underneath the object as easily as with a soft surface. While Dusty is highly effective at picking up an isolated object on a flat surface, evaluations of Dusty's performance in real environments merit further investigation, since clutter, obstacles and other common features of human environments might reduce Dusty's performance.

Participants could effectively use Dusty to fetch a TV remote control from the floor and deliver it to themselves. The participants were able to complete all but 1 of 60 trials, demonstrating the efficacy and robustness of the entire human-in-the-loop system. In addition, participants were able to safely interact with Dusty. The robot briefly made physical contact with one participant's shoe and another's thigh. However, participants did not report any discomfort or pain. Interestingly, these results suggest that contact may be likely when users teleoperate an assistive mobile manipulator. This would imply that designers should work to make contact safe or enable the robot to actively avoid contact.

On average, it took participants ∼ 1 minute to use Dusty to retrieve a dropped object from the floor. It is worth noting that the current lift takes ∼17 seconds to fully extend. We expect that future improvement on the design of the lift could reduce the retrieval time and improve the robot's performance. We also expect that users would become more proficient with the robot with practice. People often appeared to get confused when driving the robot when it was facing towards them. During real world use, the locations of the robot, obstacles, and target objects would vary, and multiple obstacles might present greater challenges for object retrieval. Obstacles might also obscure the user's view of the robot or target object. All of these factors could influence the time required to retrieve an object in the real world setting. An interesting possibility to mitigate problems with occlusions and varying perspectives while driving the robot would be to include a camera on the robot. This camera could also have lights to enable the user to operate the robot in dark environments such as underneath furniture. Undergraduates in a senior design course in bio-medical engineering at Georgia Tech prototyped a camera-based interface to teleoperate Dusty, which was very promising during informal testing. Alternatively, the robot could use autonomous navigation to find the user.

Participants reported high overall satisfaction using Dusty, and a majority of them would prefer using Dusty over their current retrieval methods (70%). Although 17 participants reported *using their hands* to retrieve dropped objects, 12 of them stated that they would prefer to use Dusty. This suggests that although they reported that they are able to use their hands, retrieving objects may still be challenging and a robot like Dusty can be still beneficial to them. This interpretation is further supported by the participants' reports that Dusty is significantly easier to use than their hands. In addition, participants rated Dusty to be significantly easier to use than *asking family members* and *using reachers*.

Several of the participants' responses indicate that they would prefer having a robot that is compact in size. Two participants who previously interacted with EL-E reported that they would prefer using Dusty to deliver an object to them because of Dusty's compact size, portability and faster time to deliver the object. Two participants would want Dusty to be 20%–30% smaller. In addition, limited space was the most common reason given for not using Dusty in any type of room. These results suggest that assistive robots may be more useful and better accepted if they are compact in size and perform tasks quickly. Our results suggest that making Dusty smaller might enable it to be used in more rooms within peoples' homes. The influence of various dimensions is less clear. The robot's footprint may be important for operating in other rooms, while the robot's height (comparing EL-E and Dusty) might be important for human–robot interaction.

We found no significant difference in task completion time between powered wheel chair (PWC) owners and participants who did not own a PWC. On a related note, we found that PWC owners had a significantly higher level of disability based on comparing their ALSFRS scores. Thus, the level of experience with the PWC joystick interface and the level of disability may be confounded, which may partially explain why performance levels were not significantly different between the two groups. Our results indicate that PWC users might benefit more from a robot like Dusty, since they reported significantly higher rates of dropping objects and reported using significantly more methods to retrieve them (Table V).

Dusty is a low-cost solution relative to other assistive mobile manipulators. We believe that through cost engineering and commercial production, the cost of this robot could be dramatically reduced. Also, Dusty could potentially be improved with more autonomy, such as using a clickable-world style of interface [35]. Compact and economical assistive mobile manipulators, like Dusty, may be able to perform a variety of other tasks, such as delivering pills, telemedicine and operating household devices. How general this class of robots can be is an open question, although the capabilities of helper monkeys and service dogs suggest that small-scale mobile manipulators could be very capable in the long run.

## Conclusion

We have developed a relatively low-cost mobile manipulator named Dusty that retrieves dropped objects for people with motor impairments. Our experiments show that Dusty is highly effective in grasping various objects on various floor types. We evaluated this robot with 20 people with ALS and the results show that they can use Dusty to robustly, safely and effectively retrieve an object from the floor. The users reported high satisfaction with Dusty and reported that the robot was significantly easier to use than their current object retrieval methods. Furthermore, a majority of the participants would prefer using Dusty over their current methods. Overall, our results suggest that a commercial robot similar to Dusty could provide valued assistance to people with physical disabilities in the near term. However, studies involving long-term real-world use would be required to assess the true potential of this emerging form of assistive technology.
